# 495. Evaluation of Antigen Testing for Detection of COVID-19 Vaccine Breakthrough Cases in Long-Term Care

**DOI:** 10.1093/ofid/ofab466.694

**Published:** 2021-12-04

**Authors:** Meghan Linder, Sarah Humphrey-King, Rebecca Pierce, Sheri L Hearn, Melissa Sutton, Shane Sevey, John Fontana, Paul Cieslak, Dat Tran

**Affiliations:** 1 Oregon Health Authority, Portland, Oregon; 2 Oregon Health Authority, Oregon State Public Health Lab, Hillsboro, Oregon

## Abstract

**Background:**

Long-term care facilities (LTCFs) are at high risk for severe COVID-19 outbreaks due to their congregate nature and vulnerable population. Oregon Health Authority (OHA) deployed point-of-care antigen (Ag) tests to promptly identify COVID-19 cases in LTCFs. However, their performance in identifying vaccine breakthrough cases has not been evaluated.

**Methods:**

During 2/25/21–5/25/21, OHA supported testing of residents and staff for two outbreaks at a single LTCF. Paired nasal swabs were collected and tested for SARS-CoV-2 by CDC Influenza SARS-CoV-2 Multiplex PCR Assay (molecular test) and Abbott BinaxNOW COVID-19 Ag Card (Ag test) twice weekly during the outbreaks. Participants were considered fully vaccinated if ≥ 14 days had passed since completion of a vaccine series; all others were deemed unvaccinated. A vaccine breakthrough case was defined as a positive Ag or molecular test from a fully vaccinated person’s specimen. Performance characteristics of the Ag test were assessed, with molecular test as the reference standard. Cycle threshold (Ct) values were compared by one-sided independent t-tests.

**Results:**

94 unvaccinated residents and staff provided 563 paired samples; SARS-CoV-2 was detected in 21 (12 by Ag and molecular test, 6 by molecular test only, 3 by Ag test only), yielding Ag test sensitivity of 66.7% (95% CI: 43.8–83.7%) and specificity of 99.4% (95% CI: 98.4–99.8%). Mean Ct values were higher for specimens positive by PCR but negative by Ag than those positive by both (30.0 vs. 20.7, *P* < .01). 81 vaccinated persons provided 925 paired samples; SARS-CoV-2 was detected in 5 (1 by Ag and molecular test, 4 by molecular test only), yielding Ag test sensitivity of 20% (95% CI: 3.6–62.5%) and specificity of 100% (95% CI: 99.6–100%). Mean Ct values for specimens from vaccinated cases were higher than those from unvaccinated cases (30.2 vs. 23.8, *P* < .05). The lone Ag-positive breakthrough case had a Ct of 20; all others had Ct > 29.

**Conclusion:**

Ag test performance and reduced sensitivity on specimens with high Ct values found in this population are consistent with published data. Molecular testing maximizes identification of vaccine breakthrough cases. More studies are needed to estimate the proportion of breakthrough cases missed by Ag testing and their risk of transmitting the virus in LTCFs.

**Disclosures:**

**All Authors**: No reported disclosures

